# The best temperature range to acquire reliable thermal infrared spectra from orbit

**DOI:** 10.1038/s41598-021-92130-1

**Published:** 2021-06-24

**Authors:** F. Nestola, S. Ferrari, M. G. Pamato, G. Redhammer, J. Helbert, M. Alvaro, M. C. Domeneghetti

**Affiliations:** 1 Department of Geosciences, University of Padua, via Gradenigo 6, 35131 Padova, Italy; 2 Center of Studies and Activities for Space (CISAS) “G. Colombo”, University of Padua, Via Venezia 15, 35131 Padova, Italy; 3 Department Chemistry and Physics of Materials, Division Material Sciences and Mineralogy, University of Salzburg, Hellbrunnerstraße 34, 5020 Salzburg, Austria; 4 German Aerospace Center (DLR), Institute for Planetary Research, Rutherfordstraße 23, 12489 Berlin, Germany; 5 Department of Earth and Environmental Sciences, University of Pavia, Via A. Ferrata 1, 27100 Pavia, Italy

**Keywords:** Planetary science, Mineralogy

## Abstract

Solar System bodies undergo to daily and periodical variations of temperature that mainly depend on their closeness to the Sun. It is known that mineral expansion and contraction due to such variations modify the thermal infrared spectra acquired on solid surfaces. Therefore, it becomes crucial to know the best temperature range at which the acquisition itself should be carried out to get reliable information on the mineralogy of such bodies. Here we provide the thermal expansion of olivine between 20 and 298 K determined by X-ray diffraction. Our data reveal the non-linear behaviour of silicates that undergo to low temperatures, where volume variations appear positively correlated with temperatures. Subtle bond-length variations occurring at low temperatures are then expected to minimally affect vibrational absorption positions. We suggest that thermal infrared spectra of those Solar-System surfaces that are not exceeding 300 K provide reliable information about not only the silicate mineral identification but also on their chemical composition, regardless of the instantaneous temperature.

## Introduction

Extreme temperature fluctuations due to daily illumination conditions affect the remote sensing response of minerals that build up Solar-System solid surfaces. Mercury, for instance, is a slow-rotation planet very close the Sun that undergoes to surface temperature ranges of hundreds of degrees^1^. Similarly, several minor rocky bodies like Near Earth asteroids experience large temperature variations during their inbound and outbound journeys around the Sun because of the great eccentricity of their orbits^2^. Such variations, which strongly affect the crystal structure of minerals, make the interpretation of remote sensing thermal infrared (TIR) spectra much more challenging. It is known, indeed, that in the middle portion of the electromagnetic spectrum (i.e. 100–5000 cm^−1^) absorption features arise by cation–anion and lattice vibrations of the crystal structure^3^. The way in which any crystal structure responds to temperature variations is by expanding or contracting^4^, therefore modifying bond lengths and energies absorbed during atom vibrations, and affecting shape and position of TIR absorption features.

The influence that the thermal expansion exerts on TIR spectra of minerals has been recently explored in support of the future employ of the BepiColombo Mercury Radiometer and Thermal Infrared Spectrometer (MERTIS)^5^, which was launched on October 20, 2018. Extended laboratory studies have been conducted on silicates^6–9^, which are the main constituents of Solar System rocky surfaces, in order to unveil their spectral behavior as a function of the daily variation of Mercury’s temperatures. In particular, it has been shown^7^ that, increasing the sample temperature from 298 to 773 K, the Si–O stretching vibration absorption bands of a Mg_1.84_Fe_0.16_SiO_4_ olivine reach markedly shifts toward lower wavenumbers, up to 8 cm^−1^. Concerning olivine, previous works investigated the effect of temperature on the cell size^10,11^ and on IR spectra^12–15^ as separate consequences. The authors^7^ investigated the unit-cell volume changes of the same Mg-rich olivine in situ, from 298 to 773 K, to correlate the olivine thermal expansion with the TIR spectra collected within the same range of temperatures. The authors confirmed that the unit-cell expansion couples with the spectrum shift. As this process is completely reversible (i.e. the volume expands upon heating and reduces back to the starting value after cooling) also the spectrum will show no signs of the heating process that has occurred. On the other hand, the same shift can be obtained without temperature variations but with a change of the Mg/Fe ratio^16^. Olivine, indeed, is an ideal solid solution between forsterite (Fo, Mg_2_SiO_4)_, and fayalite (Fa, Fe_2_SiO_4_), and thus the unit-cell volume along such binary join changes linearly^17^ substituting Mg with Fe^2+^. As reported by these authors, the unit-cell volume increases linearly with increasing the Fe content through the following equation:

1 $${\text{V }}\left( {{\AA}^{{\text{3}}} } \right){\text{ }} = {\text{ 3}}0{\text{8}}.{\text{56 }}{-}{\text{ }}0.{\text{18}}0{\text{1 }} \times {\text{ }}\% {\text{Fo}}$$

Based on Eq. (1), we could observe an increase in the unit-cell volume because of the increasing Fe-content and/or because of an increase in temperature. An increase in temperature by about 420 K causes the same increase in volume caused by an enrichment in iron from Fo_92_ to Fo_71_ (i.e. from Mg_1.84_Fe_0.16_SiO_4_ to Mg_1.42_Fe_0.68_SiO_4_), without any temperature variations^7^. Therefore, spectral variations due to thermal expansion provide a temporary mismatch between remote sensing data and those of traditional *room*-temperature databases, affecting the correct interpretation of the surface composition as much as irreversible transformations (e.g., spectral flattening due to shock impacts) affect possible structural inferences (e.g., particle size). Knowing the effect of thermal expansion on TIR spectra becomes crucial to understand what happens to the unit-cell volume of olivine in the complete low–high temperature range.

In this work, we investigate the thermal expansion of the Mg_1.84_Fe_0.16_SiO_4_ olivine sample^7^ between 298 and 20 K by means of precise and accurate X-ray powder diffraction technique. These data, combined with those provided by^7^, cover the complete range of temperatures attainable in the inner Solar System and suggest which would be the best ranges to acquire remote sensing thermal infrared spectra from bodies characterized by extremely variable surface temperatures.

In addition, this study aims to find the best temperature range in which the thermal infrared spectra of olivine should be collected by spectrometers, whether they are remote or lander instruments, on a cold silicate surface of the Solar System in order to obtain a reliable mineralogical composition. Indeed, several cold surfaces of minor bodies, as well as of Mars and the Giant Planets’ moons, retain or could potentially retain silicates.

## Results

**Figure 1:**
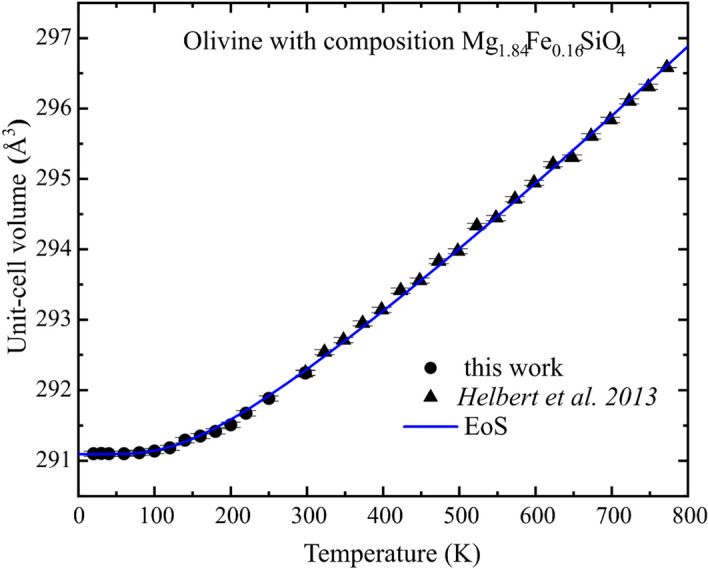
Evolution of the unit-cell volume of olivine as a function of temperature. The unit-cell volume of olivine with composition Mg_1.84_Fe_0.16_SiO_4_ is plotted versus temperature between 20 and 773 K. Triangles are from^7^ whereas circles correspond to the data collected in this study. The blue curve represents the fit of all data (see text for more details).

The good agreement between the low-T (present study) and high-T^7^ data allowed us to reliably combine the two datasets into a single one. The evolution of unit-cell volumes of our sample as a function of temperature from 298 to 20 K is reported in Table 1, and shown in Fig. 1 together with data measured from 298 to 773 K^7^. The unit-cell volumes decrease non linearly from 298 to 80 K, whereas from this temperature down to 20 K we do not observe any volume decrease higher than the experimental uncertainties. The volume variation between 298 and 80 K (a temperature variation equal to about 220 K) is only 1.13 Å^3^ (i.e. 0.39%). The high-temperature study^7^ reported between 298 and 498 K (a temperature variation equal to 200 K) an almost double volume variation. Those measurements show how different are the thermal expansion behaviors at high and low temperature conditions for olivine.

**Table 1:** Unit-cell parameters as a function of temperature.

*T* (K)	*a* (Å)	Δ*a*	*b* (Å)	Δ*b*	*c* (Å)	Δ*c*	*V* (Å^3^)	Δ*V*
298	4.76599	0.00003	10.22897	0.00007	5.99458	0.00005	292.24	0.04
250	4.76458	0.00003	10.22399	0.00007	5.99188	0.00005	291.88	0.04
220	4.76379	0.00003	10.22090	0.00007	5.99037	0.00005	291.67	0.04
200	4.76311	0.00003	10.21878	0.00007	5.98913	0.00005	291.51	0.04
180	4.76279	0.00003	10.21737	0.00007	5.98843	0.00005	291.42	0.04
160	4.76259	0.00003	10.21622	0.00007	5.98798	0.00005	291.35	0.04
140	4.76241	0.00003	10.21532	0.00007	5.98755	0.00005	291.29	0.04
120	4.76204	0.00003	10.21365	0.00007	5.98677	0.00005	291.18	0.04
100	4.76189	0.00003	10.21295	0.00007	5.98641	0.00005	291.14	0.04
80	4.76187	0.00003	10.21254	0.00007	5.98620	0.00005	291.11	0.04
60	4.76189	0.00003	10.21195	0.00007	5.98622	0.00005	291.10	0.04
40	4.76181	0.00003	10.21208	0.00007	5.98626	0.00005	291.10	0.04
30	4.76184	0.00003	10.21208	0.00007	5.98628	0.00005	291.10	0.04
20	4.76184	0.00003	10.21198	0.00007	5.98628	0.00005	291.10	0.04
298*	4.76595	0.00003	10.22904	0.00007	5.99467	0.00005	292.25	0.04

Unit-cell parameters and volumes of olivine studied in this work with composition Mg_1.84_Fe_0.16_SiO_4_ at different temperatures between 20 and 298 K.

Data are scaled to 298 K unit-cell parameters of Table 4 for the high-T dataset^7^ as the differences in unit-cell parameters were negligible. Raw low-T data are available for Mg_1.84_Fe_0.16_SiO_4_ olivine, which contains NIST silicon as internal standard.

*Measured after cooling to verify the perfect reversible behavior before and after the cooling to 20 K.

From this extended dataset, we can determine the elastic behavior of olivine in the entire range of temperature occurring on Mercury surface (i.e. from about 700 K to about 70 K). On the other hand, our dataset can be easily applied to any Solar System surface that shows extreme temperatures. However, processing such extended dataset requires a careful data analysis in order to choose the correct equation of state (hereafter EoS) to be used. Following recent reviews^18^, fitting of the extended dataset was performed using a Kroll-type EoS, available on EoSFit7 software^19,20^ to guarantee robustness of the fittings. The resulting volume thermal expansion coefficient (α_v_), room-T unit-cell volume (V_0_) and the Einstein temperature (θ_E_) values are reported in Table 2 (linear thermal expansion coefficients are reported in Table 3). The advantage of this model is that the only additional parameter is a characteristic Einstein temperature that can be estimated from entropy measurements^21^ or obtained by fitting V-T data^18^. In our study, we used the value of *K*′ equal to 4.47, which is the best estimate obtained in the recent review of the elastic properties of olivine^18^. Similar fitting strategies were used in^19,22,23^. The θ_E_ value of 450 K reported in Table 2 seems to be consistent with the values of 497(7) K for Fo_91_^18^, and with the estimate of 531 K for Fo_100_ from the measured entropy^21^ and with the decrease in θ_E_ with increasing Fe content^10^. The fit is reported in Fig. 1 along the entire low–high temperature data set^7^. Our volume thermal expansion values are in perfect agreement with previously published data at room-T and high-temperature^10^ and with a recent review on mantle olivine^24^. The value of volume thermal expansion coefficient, α_V,298_ = 2.673 10^−5^ K^−1^ (see Table 2) for the olivine studied in this work, is similar to that of other planetary rock-forming minerals like plagioclases, which show values in α_V,298_ between 1.6 and 2.8 10^−5^ K^−1^^25^, and pyroxenes for which most values of α_V_ is between 2.3 and 2.9 10^−5^ K^−1^^8,26,22^ (see Table 4). Such similarities allow us to extend the following discussion carried out for olivine to most of the silicates considered the main constituting minerals of the surface of most Solar System bodies.

**Table 2:** Thermoelastic parameters for olivine Mg_1.84_Fe_0.16_SiO_4_.

*V*_0_ (Å^3^)	292.281 (8)
α_V,298_ (10^−5^ K^−1^)	2.673 (12)
*K*′	4.47*
θ_E_ (K)	450

Thermoelastic parameters of the olivine studied in this work obtained using a Kroll-type equation of state available on EoSFit7 software^18^. The first pressure derivative *K*′ was taken from^17^.

*This value is from^17^.

**Table 3:** Linear thermal expansion coefficients of olivine Mg_1.84_Fe_0.16_SiO_4_.

Linear thermal expansion coefficients	
*α*_*a*_ [K^−1^]	0.313 × 10^−5^
*α*_*b*_ [K^−1^]	0.598 × 10^−5^
*α*_*c*_ [K^−1^]	0.499 × 10^−5^

Linear thermal expansion coefficients *α*_*l*_ [(*l*_max_ − *l*_min_)/*l*_min_]/(ΔT)] within the temperature range investigated using the data reported in Table 1.

**Table 4:** Volume thermal expansion coefficients.

Minerals	Compositions	*α*_*v*_ [K^−1^]	References
Plagioclases	(Ca,Na)(Al,Si)_2_Si_2_O_8_	1.6–2.8 × 10^−5^	^20^
Clinopyroxenes	Ca(Mg,Fe)Si_2_O_6_	2.7–2.9 × 10^−5^	^8,21^
Orthopyroxenes	(Mg,Fe)_2_Si_2_O_6_	2.31–2.51 × 10^−5^	^22^
Olivine	Mg_1.84_Fe_0.16_SiO_4_	2.67 × 10^−5^	This study

Volume thermal expansion coefficients, *α*_*v*_, for the main constituent phases of planetary bodies.

## Discussion

Our results (data between 20 and 298 K) combined with those by^7^ (data between 300 and 773 K) in Fig. 1 show how the volume of olivine displays larger volume changes at high-temperature values and smaller volume variations at low-temperature regimes. Since the shifts of the position of the TIR absorption bands strongly depend on unit-cell volume changes, greater shifts will occur at higher temperatures as well as negligible shifts will occur at very low temperatures. Such observation is extremely important in terms of spectral interpretation: it is likely that TIR spectra acquired on low-temperature surfaces would not show any observable shift between 20 and 120 K (Fig. 1, Table 1).

From 352 to 773 K^7^, the main absorption band of olivine occurring at about 940 cm^−1^ undergoes a total shift toward higher wavenumbers of 8 cm^−1^. For such temperature range, the unit-cell volume of the same sample undergoes an increase from 292.71 to 296.58 Å^3^, i.e. a positive increase of 3.87 Å^3^, which corresponds to an average increase of 0.0091 Å^3^/K if taken as linear. Instead, our new results on olivine (see Table 1) measured between 80 and 298 K show that the average increase is 0.0052 Å^3^/K (linear calculation) and becomes an even smaller volume variation between 160 and 80 K being 0.0030 Å^3^/K.

How these results could affect the TIR spectra of olivine at very low temperature values remain still unknown. However, assuming a linear behavior of the band shifts^8,16^, we could predict a proportional shift of 4.1 cm^−1^ between 298 and 80 K. We know that between 80 and 298 K the volume increase is almost halved with respect to the 352 to 773 K range, and thus it would be reasonable to expect a much smaller band shift. Based on our results, we could predict that such shift would be very small for temperature ranges between 160 and 298 K, and almost negligible between 80 and 160 K. If such prediction will be confirmed by the low-temperature TIR spectra dataset, we will be able to comprehend the thermal behavior of olivine and of any other silicates on Mercury and other Solar System bodies in order to retrieve their correct chemical composition bypassing any temperature influence.

**Figure 2:**
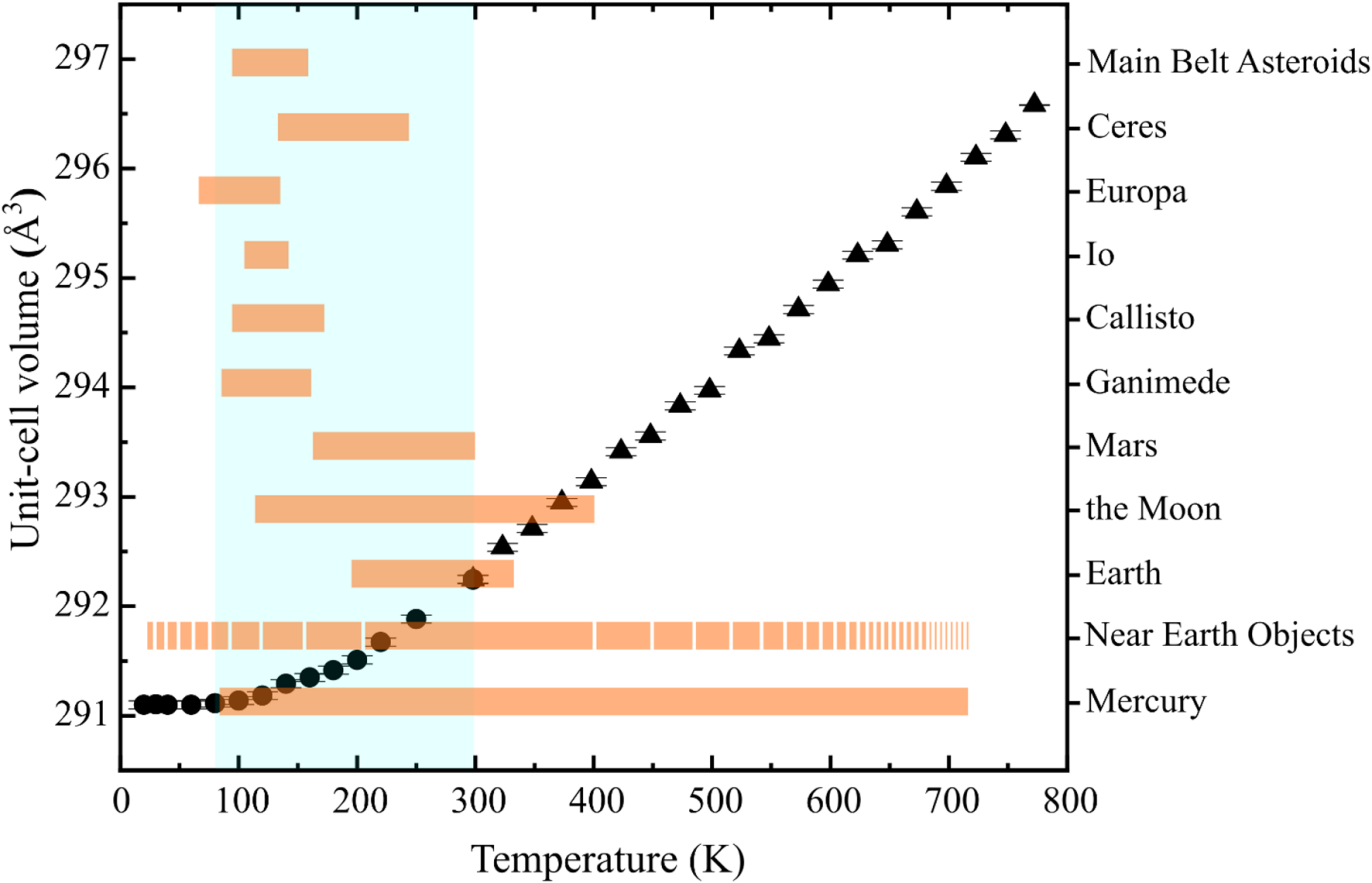
Surface temperature ranges of several Solar System bodies. The light cyan window encloses the values of volume expansion discussed in this work; orange bars show surface temperature range of several Solar System bodies that match the range discussed in this work; black circles indicate the evolution of volume as a function of temperature for olivine Mg_1.84_Fe_0.16_SiO_4_ here investigated.

A number of Solar System solid surfaces, indeed, have relatively low surface temperatures (e.g., Fig. 2) and, as well as the Mercury’s one, some of them are going to be explored by international missions. For instance, the Near Earth asteroid 1999 RQ_36_/101955 Bennu were recently globally scanned by the OSIRIS-REx Thermal Emission Spectrometer (OTES)^23^. Such orbit acquisitions reasonably depend on the signal-to-noise ratio, which partly depends on the body temperature. Acquisitions, indeed, were scheduled on the sunlit portion of the body in a well-calibrated range of temperatures, which are higher than those suggested in this work for negligible band position shifts (i.e., 80–160 K). The surface of Bennu can undergo midday temperatures between 200 and 420 K depending on the solar distance, and Planck functions having temperatures between 150 and 380 K were used to obtain OTES emissivity spectra. The average TIR spectrum of Bennu describes a radiative surface volumetrically dominated by silicates^24^, but no conclusive matches have been found yet among available aqueously-altered meteorites. Nevertheless, considering the best resolution in cm^−1^ achievable by hyperspectral instruments, we can be confident that any possible chemical composition derivable between 80 and 300 K will be not affected by thermal expansion effects.

Our analysis suggests that high-resolution TIR spectra of Main Belt asteroids, giant planet’s moons and Mars—of which surfaces do not exceed 300 K—provide reliable information about not only the silicate mineral identification but also about their chemical composition. Such information becomes critical in determining the geological environment of body evolution. On the other hand, Solar System hot surfaces like those of Mercury and most of the Near Earth asteroids would benefit of sunset TIR acquisitions as well as suitable high temperature spectra databases.

## Materials and methods

In this study, we have investigated a natural olivine^7^ with composition Mg_1.84_Fe_0.16_SiO_4_. The olivine sample is from the high-pressure ultramafic nodules from the Newer Volcanics of Mt. Leura, Victoria, Australia^25,26^. The sample has been fully characterized by single-crystal X-ray diffraction (SCXRD) and X-ray powder diffraction (XRPD)^7,25,26^ and by Electron Microprobe Analysis (EMPA)^29^.

Precise measurements of unit-cell lattice parameters and volumes were performed using step-scan powder X-ray diffraction measurements in the temperature range 20 K—300 K. Each measurement was performed in a 2θ range between 10 and 100° (continuous scan) on a Philips X'Pert diffractometer (CuKα1,2 radiation, fixed 0.125° divergence slit, primary and secondary 0.04 rad Soller slits, X'Celerator detector) using a Janis CCS-250 cryostat at the Institute of Crystallography, RWTH Aachen, Germany. Temperature stability was achieved to better than 1 K. Lattice parameters were obtained from whole diffraction pattern refinement using the FULLPROF-suite^31^ and are reported at each temperature in Table 1.

## Data Availability

All data needed to evaluate the conclusions in the paper are present in the paper.
